# Bloch–Siegert *B*_1_-Mapping Improves Accuracy and Precision of Longitudinal Relaxation Measurements in the Breast at 3 T

**DOI:** 10.18383/j.tom.2016.00133

**Published:** 2016-12

**Authors:** Jennifer G. Whisenant, Richard D. Dortch, William Grissom, Hakmook Kang, Lori R. Arlinghaus, Thomas E. Yankeelov

**Affiliations:** 1 Department of Radiology and Radiological Sciences, Vanderbilt University, Nashville, Tennessee;; 2 Vanderbilt University Institute of Imaging Science, Vanderbilt University, Nashville, Tennessee;; 3 Department of Biomedical Engineering, Vanderbilt University, Nashville, Tennessee;; 4 Department of Biostatistics and Center for Quantitative Sciences, Vanderbilt University, Nashville, Tennessee; and; 5 Institute for Computational and Engineering Sciences, and the Departments of Biomedical Engineering and Internal Medicine, The University of Texas at Austin, Austin, Texas

**Keywords:** *T*_1_ mapping, quantitative breast MRI, relaxometry

## Abstract

Variable flip angle (VFA) sequences are a popular method of calculating *T*_1_ values, which are required in a quantitative analysis of dynamic contrast-enhanced (DCE) magnetic resonance imaging (MRI). *B*_1_ inhomogeneities are substantial in the breast at 3 T, and these errors negatively impact the accuracy of the VFA approach, thus leading to large errors in the DCE-MRI parameters that could limit clinical adoption of the technique. This study evaluated the ability of Bloch–Siegert *B*_1_ mapping to improve the accuracy and precision of VFA-derived *T*_1_ measurements in the breast. Test–retest MRI sessions were performed on 16 women with no history of breast disease. *T*_1_ was calculated using the VFA sequence, and *B*_1_ field variations were measured using the Bloch–Siegert methodology. As a gold standard, inversion recovery (IR) measurements of *T*_1_ were performed. Fibroglandular tissue and adipose tissue from each breast were segmented using the IR images, and the mean *T*_1_ was calculated for each tissue. Accuracy was evaluated by percent error (%err). Reproducibility was assessed via the 95% confidence interval (CI) of the mean difference and repeatability coefficient (*r*). After *B*_1_ correction, %err significantly (*P* < .001) decreased from 17% to 8.6%, and the 95% CI and *r* decreased from ±94 to ±38 milliseconds and from 276 to 111 milliseconds, respectively. Similar accuracy and reproducibility results were observed in the adipose tissue of the right breast and in both tissues of the left breast. Our data show that Bloch–Siegert *B*_1_ mapping improves accuracy and precision of VFA-derived *T*_1_ measurements in the breast.

## Introduction

Dynamic contrast-enhanced magnetic resonance imaging (DCE-MRI) is a common method for evaluating tumor response to therapy in a variety of cancers (1–3), including breast (4, 5). DCE-MRI acquires images before, during, and after injection of a contrast agent to characterize, for example, tumor-related perfusion. To perform a quantitative analysis of DCE-MRI data, knowledge of the precontrast longitudinal relaxation time (*T*_1_) is required to convert the measured dynamic signal intensity into a time course of the concentration of the contrast agent (6). A popular technique used to measure the precontrast *T*_1_ is the variable flip angle (VFA) approach, which uses a series of spoiled gradient echo (SPGE) images acquired with a short, fixed repetition time (TR) and a varying flip angle (7, 8). The resulting data are then fit to the signal intensity equation describing the SPGE acquisition with *T*_1_ as a fit parameter for each voxel or region of interest (ROI). Although this technique allows for rapid 3-dimensional (3D) *T*_1_ mapping, it is not without limitations, chief of which is that its accuracy is dependent on the uniformity of the transmit radiofrequency (*B*_1_) field. It should be noted that other *T*_1_ mapping methods exist that are less sensitive to variations in the transmit field (9); however, VFA sequences are the preferred method in the clinical setting, as these acquisitions enable a large field of view (FOV) to be measured in a relatively short period.

Inhomogeneities in the *B*_1_ field cause variations in the prescribed flip angles, leading to inaccurate measurements of *T*_1_, which can subsequently induce large errors in the DCE-MRI parameters (eg, the volume transfer rate constant, *K^trans^*) (10). Indeed, simulation results indicated that errors in *K^trans^* ranged from 15% to 500% as the error in the *T*_1_ measurement ranged from 14% to 65% of the nominal value (11). Therefore, an inaccurate estimation of the precontrast *T*_1_ could potentially lower the sensitivity of DCE-MRI for characterizing tumor vascular properties, thereby limiting the utility of the technique.

The *B*_1_ field experienced by spins within the body is influenced by several factors, including the distance of the spins from the radiofrequency transmit coil, the dielectric properties of the tissues, and the factors related to body size and wavelength of the radiofrequency (12). The severity of the nonuniformity in the *B*_1_ field increases at higher field strengths (13), and noticeable *B*_1_ inhomogeneities have been observed in the breast at 3 T (14–17). In particular, a substantial variation in the *B*_1_ field from left to right across the imaging FOV has been observed, which may artificially decrease the contrast enhancement in specific lesions (18). Thus, Kuhl et al. have suggested that *B*_1_ mapping in the breast should be a standard practice (14). Although several different methods for *B*_1_ mapping have been developed (12, 19–22), no single method has emerged for widespread application.

A technique using the Bloch–Siegert shift to map the *B*_1_ field has recently been developed, and it is an area of active investigation (23–26). The Bloch–Siegert shift is a term used to describe the shift in resonance frequency of a nucleus when an off-resonance radiofrequency field is applied (27). Although Sacolick et al. (24) provide the details, the salient information is mentioned here. If a radiofrequency pulse is applied either far enough off-resonance and/or with a pulse shape such that it does not cause spin excitation, the spins experience a change in precession frequency without excitation (28). The spin precession frequency shifts away from the off-resonance irradiation and is dependent on the magnitude of the *B*_1_ field, as well as the difference between the spin resonance frequency and radiofrequency field. The shift in frequency results in a phase shift in the images that can be used to spatially map the *B*_1_ magnitude. This phase-based method generates a *B*_1_ map that is not significantly biased by TR, *T*_1_ relaxation, flip angle, chemical shift, background field inhomogeneity, or magnetization transfer (24). The insensitivity to TR is especially important in a clinical setting, as it allows for the prompt acquisition of image data with a short TR. In the current study, we present an approach to rapidly, accurately, and precisely map *B*_1_ and *T*_1_ values in the breast using the Bloch–Siegert method with a VFA sequence.

## Methodology

All imaging data were acquired with a 3 T Achieva magnetic resonance scanner (Philips Healthcare, Best, The Netherlands) equipped with a 2-channel multitransmit body coil and a MammoTrak table including a 16-channel receive double-breast coil (Philips Healthcare, Best, The Netherlands).

### Phantom Scans

To investigate the feasibility of the approach, 8 gel phantoms (The Eurospin II Test System, Diagnostic Sonar, Livingston, Scotland, UK) submerged in water were scanned at room temperature. The *T*_1_ values of the phantoms ranged from 300 to 1600 milliseconds. A coronal image volume was placed in the center of the breast coil containing the phantoms, and VFA, Bloch–Siegert, and inversion recovery (IR) data were collected. VFA data with 10 flip angles (2, 4, 6, . . . 20) were acquired using a 3D SPGE sequence with the following parameters: TR/echo time (TE) = 7.9/4.6 milliseconds, sensitivity encoding parallel imaging factor of 2, acquisition matrix of 192 × 192 over a FOV of 256 × 256 mm^2^, yielding a voxel size of 1.33 × 1.33 mm^2^, and 15 slices with a thickness of 4 mm for a total scan time of 66 seconds. The Bloch–Siegert data were collected using a gradient echo sequence with a 2-millisecond frequency-swept *B*_1_ phase imparting pulse (25) over the same FOV as the VFA data with the following parameters: TR/TE = 491/5.4 millisecond, acquisition matrix of 104 × 102, reconstruction voxel size of 1.33 × 1.33 mm^2^, and root mean square *B*_1_ field = 2.29 μT for a total scan time of 104 seconds. As is required with the Bloch–Siegert *B*_1_ mapping, 2 images were collected at opposite frequency offsets. As a gold standard, a 2-dimensional IR-prepared turbo-spin echo (IR-TSE) sequence was used to acquire a single slice corresponding to the center of the VFA image volume with the following parameters: 12 inversion times of 25, 50, 75, 100, 200, 300, 400, 500, 1000, 2000, 4000, and 10 000 milliseconds, acquisition matrix of 128 × 96 over an FOV of 256 × 256 mm^2^, reconstruction voxel size of 1.33 × 1.33 mm^2^, predelay before inversion pulse of 2500 milliseconds, and TSE factor of 24 with an echo spacing of 5.9 milliseconds for a total scan time of 125 seconds.

### Subject Scans

Test–retest MRI sessions were performed on 16 women (median: 42 years, range: 25–67) with no history of breast disease. Because of age, body habitus, or hormonal status, 4 of these women did not have appreciable fibroglandular tissue (FGT) in either breast; thus, measurements for these women included data only from the adipose tissue (AT). The imaging protocol consisted of 2 scan sessions each lasting ∼30 minutes separated by a 10-minute rest period. During the rest period, the subjects were removed from the scanner and allowed to stretch. All subjects were consented as part of an ongoing study approved by the local Institutional Review Board.

For each test–retest session, 2 separate sagittal imaging volumes were centered on each breast with an attempt to approximately match the stack placement between each imaging session. Subsequent VFA, Bloch–Siegert, and IR data were then collected separately for each breast. The imaging parameters for each sequence were identical to the phantom scans, except that the slice thickness was 5 mm and the number of slices for the VFA and Bloch–Siegert sequences was 10. These 2 parameters were changed to match our ongoing clinical imaging trial (29). In addition, we applied the *T*_1_ and Bloch–Siegert *B*_1_ mapping methods described herein on 3 patients with breast cancer. Each patient provided written consent to participate in the study.

### Image Analysis

All image data were exported to MATLAB R2013b (The MathWorks, Natick, Massachusetts) for analysis. Bloch–Siegert *B*_1_ maps were calculated as described previously (25). In brief, the actual flip angle at each image voxel was obtained via linear interpolation of the entries of a phase difference-versus-*B*_1_ strength lookup table generated using Bloch equation simulations of the off-resonant Bloch–Siegert pulse. The flip angle correction map was then calculated as the ratio of the actual flip angle to the prescribed flip angle.

VFA *T*_1_ maps with and without *B*_1_ correction were obtained by fitting signal intensity (*S*) data to equation 1 as follows:

1 $$S=S_{0}\cdot \frac{\sin(f\cdot \alpha )\cdot (1-\exp(-TR/T_{1})}{1-(\exp(-TR/T_{1})\cdot \cos(f\cdot \alpha ))}$$

where *S*_0_ is a constant related to scanner gain and proton density, α is the prescribed flip angle, and *f* is the Bloch–Siegert-derived flip angle correction factor (set to 1 for the uncorrected *T*_1_ map), and we have taken TE ≪ *T*_2_*. In addition, *T*_1_ maps were calculated by fitting the IR-TSE data (30) to equation 2 as follows:

2 $$S=S_{0}\cdot |(\cos\alpha \cdot (1-\exp(-TD/T_{1}))\cdot \exp(-TI/T_{1}))+1-\exp(-TI/T_{1})|\cdot \;\;\;$$

For the phantom scans, circular ROIs were manually drawn within each gel phantom using the IR data as a guide. The average *T*_1_ from each phantom was recorded from the IR *T*_1_ map. The same ROIs were then subsequently used to calculate the average *T*_1_ from the central slice of the VFA-derived *T*_1_ maps with and without *B*_1_ correction. Statistical analyses were performed on the average *T*_1_ values calculated from each ROI. To evaluate the accuracy of the proposed *B*_1_ mapping technique, the percent error (%err) between the IR- and VFA-derived *T*_1_ values (with and without *B*_1_ correction) was calculated.

For healthy volunteers, segmentation masks for AT were automatically generated from the IR data (inversion times = 500 milliseconds), where the signal intensity for the FGT was close to 0. A representative example of the segmentation masks for each tissue is presented in Supplemental Figure 3. FGT segmentation masks were subsequently generated as the opposite of the AT mask after manually segmenting the skin and chest wall from the FOV. The average *T*_1_ from each tissue segmentation mask was recorded from the IR *T*_1_ maps for each breast and imaging session. The same tissue masks were then used to calculate the average *T*_1_ from the central slice of the VFA-derived *T*_1_ maps (with and without *B*_1_ correction). The %err between the VFA-derived *T*_1_ values (with and without *B*_1_ correction) and the IR *T*_1_ values was calculated to evaluate the accuracy, and the agreement between the different *T*_1_ values was assessed via the concordance correlation coefficient (CCC). Furthermore, the bootstrap 95% confidence interval (CI) of the mean differences in absolute deviation between IR- and VFA-derived *T*_1_ values (with and without *B*_1_ correction) was computed as previously described (31) using equation 3 as follows:

3 $$avg_{i}=\text{average}(|VFA\text{-}IR\text{|-|}VFA_{B_{1}}\text{-}IR|)$$

where *VFA* and *VFA*_*B*_1__ are VFA-derived *T*_1_ values without and with *B*_1_ correction, respectively. Equation 3 is first computed with all *T*_1_ values (ie, 2 scan sessions per subject equals 32 and 26 *T*_1_ values for AT and FGT, respectively, in each breast). Next, the *n* × *m* matrix of data is randomly resampled with replacement from the original data set (such that data from a subject(s) could be included more than once) and then equation 3 is recomputed. In the AT case, for example, the *m* × *n* matrix size is 16 × 2, as there are 16 patients with 2 data points each. This process is repeated 1000 times, generating a new matrix of *m* × *n* data points, which are then used to calculate the upper and lower bounds of the bootstrap 95% CIs. The number of data points *n* is the total number of subjects.

To illustrate the application of the *T*_1_ and *B*_1_ mapping techniques described herein, manual ROIs were drawn in the AT, FGT (if appreciable), and tumor of 3 patients with breast cancer. Average *T*_1_ from each ROI was calculated, and the %err between the VFA-derived *T*_1_ values (with and without *B*_1_ correction) and the IR *T*_1_ values was compared.

### Reproducibility Statistics

Reproducibility statistics used in this test–retest study follow the methods previously described by Bland and Altman (32) and are similar to what was previously implemented in the breast of healthy volunteers (33, 34). First, the difference, *d*, was calculated between the 2 VFA *T*_1_ data sets obtained for each subject and then the distribution of those differences was tested for normality using the Shapiro–Wilk test. The Kendall's Tau test was used to ensure that the magnitude of the difference values was not correlated with the parameter mean of the repeated measurements. The Wilcoxon signed-rank test was used to test the null hypothesis of no bias (ie, average difference is 0) between repeated measurements.

The statistical measurements of reproducibility were calculated as follows:

(1) The root-mean-square deviation (*rMSD*) is computed using the differences, *d*, as follows:
  4 $$r\text{MSD}=\sqrt{\frac{\sum d^{2}}{n}}.$$
(2) The 95% CI for a group of *n* subjects is shown as follows:
  5 $$\text{CI}\;=\;\pm \frac{1.96\cdot \text{std}(d)}{\sqrt{n}}$$
  where std(*d*) is the standard deviation of *d*. The confidence interval indicates the range of expected measurement variability in a group of *n* subjects.
(3) The within-subject standard deviation (*wSD*) is as follows:
  6 $$\text{wSD}=\frac{\text{rMSD}}{\sqrt{2}}$$
(4) The repeatability coefficient (*r*) is shown as follows:
  7 r = 2.77⋅wSD

Or, equivalently, as follows:

8 r = 1.96⋅rMSD

The repeatability coefficient defines the magnitude of the maximum difference expected in 95% of paired observations; for example, *r* defines the expected measurement variability for an individual. Because of our moderate sample sizes, we replaced 1.96 in equation 5 with the appropriate *t*-statistic for our sample size, which was 2.131 (n = 16) and 2.179 (n = 13) for AT and FGT, respectively.

Statistical analyses were performed using the statistical toolbox in MATLAB^®^. A significance value of *P* ≤ .05 was used for all statistical tests. In addition, we quantified the coefficient of variation (CV) between the repeated measurements, which is the ratio of the standard deviation of the repeated measurements over the mean of the repeated measurements.

## Results

### Gel Phantoms

**Figure 1.**
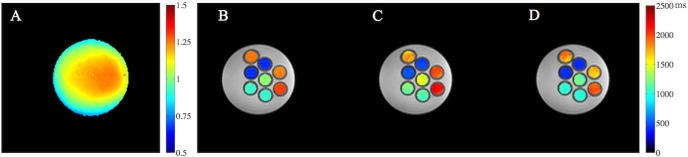
A Bloch–Siegert *B*_1_ map (A) with *T*_1_ parametric maps calculated from inversion recovery (IR) (B) and uncorrected variable flip angle (VFA) (C), and *B*_1_-corrected VFA data (D) are shown for the gel phantoms. The Bloch–Siegert *B*_1_ map shown is the correction factor, which is the ratio of the actual and nominal flip angles. Note the large spatial variation across the *B*_1_ map; this variation is a representation of the *B*_1_ inhomogeneity across the imaging field of view (FOV). After *B*_1_ correction, the VFA-derived *T*_1_ values (D) are more similar to the IR *T*_1_ values (B) in each gel phantom.

A set of image data for the gel phantom experiment is displayed in Figure 1. The Bloch–Siegert *B*_1_ map is displayed in Figure 1A, where each voxel represents the Bloch–Siegert-derived flip angle correction factor (ie, ratio of the actual flip angle to the nominal flip angle) that is incorporated into equation 1. *T*_1_ parametric maps generated from the IR, uncorrected VFA, and *B*_1_-corrected VFA data are displayed in panels B, C, and D, respectively, of Figure 1. The mean (±standard deviation) *T*_1_ value for each gel phantom ROI, along with the %err from the IR data, is listed in Table 1. Compared with the uncorrected VFA data, the *B*_1_-corrected VFA-derived *T*_1_ estimates have a significantly lower %err (*P* = .016, Wilcoxon signed-rank).

**Table 1.** Average ± Standard *T*_1_ (milliseconds) Values and %err in the Gel Phantoms

Tube No.	IR	VFA w/o *B*_1_	%err	VFA w/*B*_1_	%err
1	322 ± 25	364 ± 14	13	319 ± 8	1
2	328 ± 37	401 ± 10	22	331 ± 9	1
3	835 ± 37	889 ± 39	7	816 ± 20	2
4	843 ± 39	963 ± 40	14	822 ± 22	3
5	1004 ± 51	1160 ± 21	15	940 ± 43	6
6	1478 ± 54	1573 ± 59	6	1357 ± 56	8
7	1500 ± 19	1412 ± 64	6	1454 ± 87	3
8	1558 ± 22	1727 ± 62	8	1558 ± 50	3

Abbreviations: No., number; %err, percent error; IR, inversion recovery; VFA, variable flip angle.

### In Vivo Scans

**Figure 2.**
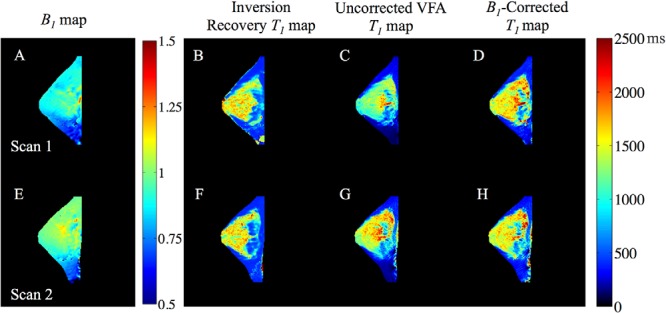
A representative test–retest set of *B*_1_ and *T*_1_ parametric maps displayed for the right breast of a healthy volunteer. Bloch–Siegert *B*_1_ maps (A and E) correspond to the correction between the actual and the nominal flip angles. Note the spatial variation of the correction factors in the *B*_1_ maps and the difference in *B*_1_ maps between repeated scans; together, these images provide evidence that a *B*_1_ map should be incorporated into routine breast imaging if a quantitative analysis of the collected data is desired. *T*_1_ parametric maps include: IR maps (B and F), uncorrected VFA maps (C and G), and *B*_1_-corrected VFA maps (D and H). The spatial variations in *T*_1_ of the FGT are minimized after *B*_1_ correction, and the *T*_1_ map more closely matches the IR *T*_1_ map. Furthermore, the *B*_1_-corrected *T*_1_ maps are visually more similar between repeated measurements compared with the uncorrected data. Observe how the orientation is slightly different between repeated scans, which might negatively affect the *T*_1_ reproducibility, as the same tissue sections from each scan might not be analyzed.

A representative set of image data is displayed for the right breast of 1 subject in Figure 2. (Identical data for the left breast in the same subject are displayed in Supplemental Figure 1.) The Bloch–Siegert *B*_1_ maps from both scans are displayed in Figure 2, A and E, where each voxel displays the Bloch–Siegert-derived flip angle correction factor. Also shown, are test–retest *T*_1_ parametric maps generated from the IR (panels B and F), uncorrected VFA (panels C and G), and *B*_1_-corrected VFA (panels D and H) data. Average *T*_1_ values from each tissue ROI and *T*_1_ mapping technique are tabulated for the right and left breast from each subject in Supplemental Tables 1 and 2, respectively.

Table 2 lists the accuracy results for each ROI and breast. In the right breast, %err in the FGT using the VFA method significantly (*P* < .001, Wilcoxon signed-rank) decreased from 17.0% to 8.6% and the CCC increased from 0.55 to 0.83 after *B*_1_ correction. Similar trends in accuracy were observed in the AT (Table 2). Bootstrap 95% CIs for FGT and AT were 57.8–139 milliseconds and 17.2–42.2 milliseconds, respectively. The range of CIs for each tissue includes all positive numbers, and by referring to equation 3, it can be seen that the absolute difference from the gold standard IR *T*_1_ is smaller after *B*_1_ correction for both tissue ROIs. In the left breast, %err in the FGT using the VFA method significantly (*P* = .002, Wilcoxon signed-rank) decreased from 15.0% to 8.7% and the CCC increased from 0.60 to 0.83 after *B*_1_ correction. Similar trends in accuracy were observed in the AT (Table 2). The bootstrap 95% CIs for FGT and AT were 35.8–104.8 milliseconds and 2.4–26.7 milliseconds, respectively; again, both values for each CI were positive, indicating that the absolute difference from gold standard IR *T*_1_ is smaller after *B*_1_ correction for both ROIs.

**Table 2.** Accuracy Results for Both Breasts and ROI

	Right Breast		Left Breast	
	%err (Std)	CCC	%err	CCC
**Adipose Tissue**				
VFA	13% (9.7%)	0.26	13% (11%)	0.29
VFA w/*B*_1_	6.2% (4.8%)	0.59	9.4% (7.3%)	0.5
**Fibroglandular Tissue**				
VFA	17% (9.1%)	0.55	15% (11%)	0.6
VFA w/*B*_1_	8.6% (7.4%)	0.83	8.7% (5.5%)	0.83

Abbreviations: ROI, region of interest; %err, percent error; Std, standard deviation; CCC, concordance correlation coefficient; VFA, variable flip angle.

**Figure 3.**
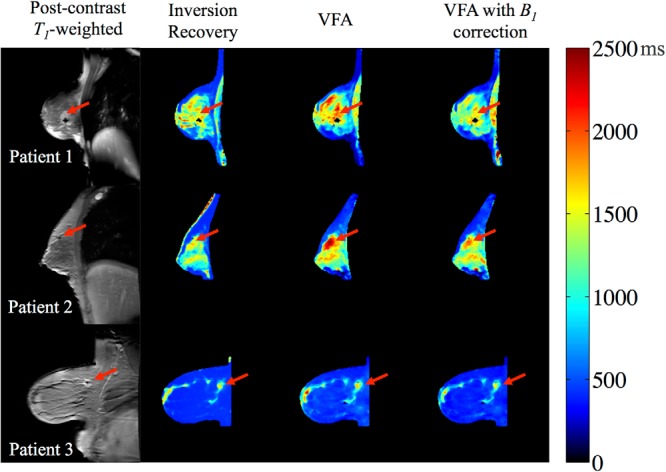
As a proof of principle, the *B*_1_ and *T*_1_ methods presented in this article were performed on 3 patients with breast cancer; each patient was a subject enrolled in our ongoing breast imaging clinical trial (29). *T*_1_ parametric maps are shown for IR (left center column), uncorrected VFA (right center column), and *B*_1_-corrected VFA (right column) data collected from each patient (shown in rows). The tumors are shown with red arrows in each image. Compared with the uncorrected VFA data, the *B*_1_-corrected VFA *T*_1_ values of the FGT, AT, and tumor in all 3 patients are more similar to the IR *T*_1_ values, thus suggesting a more accurate *T*_1_ value in each tissue after *B*_1_ correction. Note that circular regions in the breasts that have a lack of signal intensity are due to the presence of a breast biopsy clip.

As a proof of principle, the *T*_1_ and *B*_1_ mapping methods were applied in 3 patients with breast cancer. Figure 3 displays *T*_1_ parametric maps for all 3 patients generated from IR (left column), uncorrected VFA (center column), and *B*_1_-corrected VFA (right column) data. From these images, it can be seen that the *B*_1_-corrected *T*_1_ values in the tumors more closely match the IR *T*_1_ values. This similarity is extremely important, as accurate *T*_1_ values are required when performing a quantitative DCE-MRI analysis. The mean (±standard deviation) *T*_1_ value for each tissue ROI, along with the %err from the IR data, is listed in Table 3 for each patient with breast cancer. Compared with the uncorrected VFA data, the *B*_1_-corrected VFA-derived *T*_1_ estimates have, on average, a lower %err. Combining all tissue ROIs for each imaging technique, a significantly lower %err was observed after *B*_1_ correction (*P* = .004, Wilcoxon signed-rank).

**Table 3.** Average ± Standard *T*_1_ (milliseconds) Values and %err from ROIs in Patients with Breast Cancer

Patient	ROI	IR	VFA w/o *B*_1_	%err	VFA w/*B*_1_	%err
1	Tumor	1364 ± 292	1682 ± 330	23	1514 ± 269	11
	AT	411 ± 10	470 ± 48	14	384 ± 36	7
	FGT	1391 ± 336	1807 ± 215	30	1383 ± 164	1
2	Tumor	1374 ± 376	2104 ± 267	53	1721 ± 209	25
	AT	397 ± 10	439 ± 34	11	409 ± 33	3
	FGT	1493 ± 224	1725 ± 99	16	1578 ± 79	6
3	Tumor	1101 ± 345	1275 ± 381	16	1151 ± 344	5
	AT	407 ± 7	465 ± 45	14	415 ± 41	2
	FGT	1471 ± 319	2036 ± 343	38	1594 ± 271	8

Abbreviations: ROI, region of interest; %err, percent error; IR, inversion recovery; VFA, variable flip angle; AT, adipose tissue; FGT, fibroglandular tissue.

### Reproducibility

Reproducibility statistics for each tissue are listed in Table 4 and Supplemental Table 3 for the right and left breast, respectively. Normality was assumed for each data set as determined by the Shapiro–Wilk test. No data sets had an average difference significantly different from 0 as determined by the Wilcoxon signed-rank test. In addition, the Kendall's Tau test showed that the difference between repeat measurements *d* was independent of the mean for each ROI.

**Table 4.** Reproducibility Results for the Right Breast

	Mean	Mean Difference	95% CI for Mean Difference	wSD	Repeatability	CV (Mean ± SD)
**Adipose Tissue**						
VFA	429	40	±28 (6.5%)	38	104	6.7% ± 6.1%
VFA w/*B*_1_	418	19	±14 (3.3%)	18	48	3.2% ± 3.0%
**Fibroglandular Tissue**						
VFA	1316	106	±94 (7.1%)	100	276	4.7% ± 4.9%
VFA w/*B*_1_	1256	49	±38 (3.0%)	40	111	2.6% ± 1.5%

Abbreviations: CI, confidence interval; wSD, within-subject standard deviation; CV, coefficient of variation; SD, standard deviation; VFA, variable flip angle.

**Figure 4.**
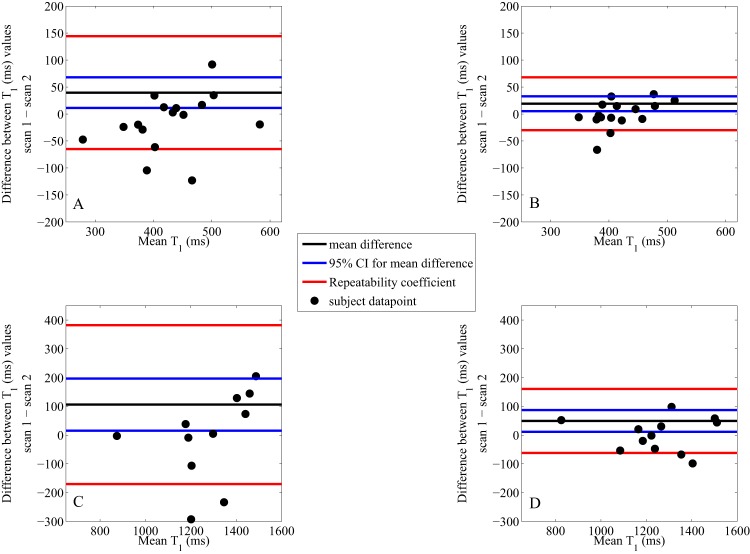
Bland–Altman plots for the right breast displaying the difference in *T*_1_ between repeated measurements plotted against mean *T*_1_ for AT before *B*_1_ correction (A), AT after *B*_1_ correction (B), FGT before *B*_1_ correction (C), and FGT after *B*_1_ correction (D). The mean difference (black line) is shown with 95% confidence intervals of the mean difference (blue lines), which defines a measure of the spontaneous variability that is expected in a cohort of subjects. Repeatability is also shown (red lines), which quantifies the maximum difference expected to be observed between 2 repeat measurements in an individual. It can be noted from the figure that the width of both the 95% CIs of the mean difference and repeatability coefficient decreases after *B*_1_ correction, suggesting a lower variability.

Bland–Altman plots for each tissue ROI are displayed in Figure 4 and Supplemental Figure 2 for the right and left breast, respectively. Each panel displays the difference in *T*_1_ between the repeated scans against the mean *T*_1_ from both scans. The mean difference and 95% CIs of the mean difference are displayed as black and blue lines, respectively. The 95% CIs of the mean difference, which define expected measurement variability for a cohort of subjects, decreased after *B*_1_ correction. For the right breast, the 95% CI of the mean difference of the AT ROI decreased from ±28 milliseconds (6.5%) to ±14 milliseconds (3.3%) after *B*_1_ correction, whereas the 95% CI of the mean difference for the FGT ROI decreased from ±94 milliseconds (7.1%) to ±38 milliseconds (3.0%). The repeatability coefficient (red lines in Figure 4 and Supplemental Figure 2), which defines the measurement variability in an individual, decreased from 104 to 48 milliseconds in AT and from 276 to 111 milliseconds in FGT after *B*_1_ correction. Similar trends in the 95% CI of the mean difference and *r* were observed for both tissues in the left breast (Supplemental Table 3).

In the right breast, the CV (Table 4) significantly (*P* = .039, Wilcoxon signed-rank) decreased from 6.7% to 3.2% in the AT after *B*_1_ correction. In the FGT, the CV decreased from 5.5% to 2.2% after *B*_1_ correction; however, the difference was not statistically significant (*P* = .064). In the left breast (Supplemental Table 3), the CV significantly decreased from 7.5% to 3.9% in the AT (*P* = .002) and 6.8% to 2.4% in the FGT (*P* = .016) after *B*_1_ correction.

## Discussion

It is well known that variations in the *B*_1_ transmit field exist in the breast at 3 T (14, 35). Thus, applying a *B*_1_ correction scheme is critical—especially when measuring *T*_1_ with an acquisition technique that requires multiple flip angles (ie, the VFA technique). Any bias in the prescribed flip angle will lead to inaccuracies in the measured *T*_1_. The observed *T*_1_ values in the AT and FGT in this study are not unreasonable and are similar to a recent study by Bedair et al. that investigated the effect of a Bloch–Siegert *B*_1_ correction technique on VFA-derived measurements of *T*_1_ in the breast at 3 T (36). In addition, our study incorporated a comparison with the gold standard IR data and a reproducibility analysis, allowing for an evaluation of accuracy and precision of the combination of the Bloch–Siegert *B*_1_ and the VFA *T*_1_ mapping techniques. We showed that the *T*_1_ and *B*_1_ mapping methods described herein are not only appropriate for clinical applications but also produce accurate estimates of *T*_1_ in breast tissues, including FGT, AT, and breast cancer.

The feasibility of the presented *T*_1_ and *B*_1_ mapping techniques was shown in the gel phantom experiment. After *B*_1_ correction, the VFA-derived *T*_1_ values in each gel phantom more closely matched the gold standard IR *T*_1_ values, which was supported by the significantly lower %err (*P* = .016, Wilcoxon signed-rank). In addition, we observed that the Bloch–Siegert *B*_1_ mapping technique improved the accuracy of the VFA-derived *T*_1_ measurements in the breast. The %err in both ROIs (ie, FGT and AT) decreased after *B*_1_ correction for both breasts, suggesting that a smaller difference exists between the *B*_1_-corrected VFA and IR *T*_1_ values as compared to the uncorrected VFA data. The bootstrap 95% CIs were positive for all ROIs (including both breasts), indicating that the *T*_1_ values after *B*_1_ correction are more similar to the IR *T*_1_ data. Furthermore, the CCC increased by ∼50% for all measurements after *B*_1_ correction. Although the CCC value in the AT ROIs increased after *B*_1_ correction, the level of agreement after *B*_1_ correction was minimal (ie, CCC = ∼0.5) and much lower than the CCCs in the FGT. The radiofrequency pulses for the Bloch–Siegert technique described in this study were designed to produce pure phase shifts over a ±600 Hz range (25); however, these phase shifts will have some (albeit small) sensitivity to off-resonance effects over that range. In principle, ±600 Hz should be sufficient for AT alone, but it could be problematic if a chemical shift exists in the field gradients. This could explain the observed lower agreement, as measured by the CCC, between the *B*_1_-corrected VFA and IR *T*_1_ values in the AT. The potential off-resonance effects should not be considered a limitation to the Bloch–Siegert method, however, and can be compensated for using a map of the static magnetic field (ie, a B_0_ map).

There have been several recent studies investigating various *B*_1_ correction schemes for accurate *T*_1_ mapping of the breast at 3 T. Sung et al. (35) evaluated the accuracy of *T*_1_ measurements in the AT using the double-angle method of *B*_1_ mapping, which is a technique that uses the signal magnitude images at nominal flip angles α and 2α. Their results showed an average relative flip angle variation of 115% on the left breast and 82% on the right breast, which improved to 7% after *B*_1_ correction (35). Although the double-angle method generates robust measurements of *B*_1_ inhomogeneity, it is limited by its *T*_1_ dependence and the requirement for long TRs to mitigate the *T*_1_ dependence, which provides a possible barrier to clinical applications. The same group developed a technique to simultaneously map *B*_1_ and *T*_1_ using the AT as a reference region, and compared their results to the double-angle method (17). This technique uses a 2-point Dixon algorithm (37) to generate AT-only images and then assigns a known *T*_1_ value to a ratio of signal magnitudes to compute the *B*_1_ field variation. Sung et al. observed that the *B*_1_ maps generated with their postprocessing technique were similar to the double-angle method (17); therefore, they concluded that their approach, which is more time-efficient than the double-angle method, could be used to correct *B*_1_ inhomogeneities in breast MRI data.

Pineda et al. also developed a reference region technique to map the *B*_1_ transmit field using a population-average *T*_1_ value in the AT that was measured using an inversion recovery spectroscopic technique (16). These investigators evaluated their *B*_1_-mapping technique by comparing VFA-derived *T*_1_ values (before and after *B*_1_ correction) to IR *T*_1_ values in the breasts of 4 patients. Before correction, the absolute difference between VFA and IR values was 58% ± 21%, which was reduced to 8.1% ± 7.8% after the *B*_1_ correction (16). Although we observed similar results in our study, the Bloch–Siegert technique described herein is not limited by the necessary assumptions of a reference region technique, with the first assumption being that the *T*_1_ of AT in the breast is globally uniform and well characterized (16, 17). The second is the requirement for a large section of tissue in the FOV with a homogenous *T*_1_, which may not always be available in, for example, women with dense breasts (17). Another *B*_1_ mapping technique that may show promise in breast imaging is the DREAM approach by Nehrke and Bornert (38), which is a novel approach for robust, ultrafast, multislice *B*_1_ mapping.

The reproducibility analysis performed in this study provides objective statistical thresholds that define the range of repeatability by quantifying the maximum difference expected to be observed between 2 repeat measurements in an individual. In addition, the 95% CIs for the mean difference provide a measure of the spontaneous variability that is expected in a cohort of subjects. Both the 95% CIs of the mean difference and repeatability coefficient are useful when defining the associated variability in a measurement so that future studies can be designed and statistically powered appropriately. We observed lower 95% CIs of the mean difference and repeatability coefficients after *B*_1_ correction in all ROIs (including both breasts). We also observed an ∼50% reduction in the coefficient of variation between the repeated measures, thus suggesting lower variability after *B*_1_ correction. Therefore, our reproducibility analysis showed that the Bloch–Siegert *B*_1_ mapping technique improved reproducibility, thereby also improving precision, of VFA-derived *T*_1_ measurements in the breast at 3 T.

We attempted to be as consistent as possible when positioning each subject in the scanner and determining the imaging FOVs between repeated acquisitions. However, image registration between repeat acquisitions was not performed because the success of the registration results would be limited by the single-slice IR acquisition. We note that this is a limitation in the current study, as tests for accuracy and reproducibility were performed using only 1 slice. We would expect, however, that applying a longitudinal registration technique (39) would only improve accuracy and precision, as differences in subject position and imaging FOV would only minimally impact the results. In addition, by using the average *T*_1_ value from all of the AT and FGT voxels in the FOV, we felt that the accuracy and precision results would not be biased by a reader who, for example, would manually draw ROIs in the tissues. Another limitation to our study is the different number of data sets in the AT and FGT analyses. Our goal was to recruit a cohort of subjects with an age range that was representative of our ongoing clinical breast imaging study (29); however, some of the women included in this study had very little or no FGT because of either age or body habitus. We noted above that off-resonance effects could limit the accuracy of the Bloch–Siegert approach in areas of the breast where a chemical shift exists in the field gradients, which, for example, could be in the AT and areas of the breast with a mixture of AT and FGT. This limitation, however, would only affect the accuracy results described herein and should not be considered as a limitation of the Bloch–Siegert method, as chemical shift effects can be minimized by incorporating a map of the static magnetic field into the correction scheme.

## Conclusion

The VFA technique is often used in clinical applications of DCE-MRI, as it allows for 3D *T*_1_ mapping in a time-efficient manner. However, the accuracy of the technique is severely affected by inhomogeneities in the *B*_1_ transmit field, which are known to be significant in the breast at 3 T (14). The difference in the prescribed flip angle due to *B*_1_ inhomogeneities leads to inaccuracies in VFA-derived estimates of *T*_1_, which can compound to large errors in, for example, the DCE-MRI parameter *K^trans^* (11, 36). Large errors in DCE-MRI analyses could lower the sensitivity and specificity of the imaging technique, thereby limiting clinical adoption. Applying a *B*_1_ correction map is 1 technique, among others, that can be used to compensate for the inhomogeneities in the transmit field. In this study, we showed that *B*_1_ correction using the Bloch–Siegert shift is a viable (and attractive) option to measure accurate and precise VFA-derived *T*_1_ values in the breast at 3 T.

### Supplemental Materials

Supplemental Figure 1:

Supplemental Figure 2:

Supplemental Figure 3:

Supplemental Table 1:

Supplemental Table 2:

Supplemental Table 3:
