# Supplementary material for: Bloch–Siegert B1-Mapping Improves Accuracy and Precision of Longitudinal Relaxation Measurements in the Breast at 3 T
Source: Tomography. 2016 Dec;2(4):250–9. doi: 10.18383/j.tom.2016.00133 (PMC5201175; doi:10.18383/j.tom.2016.00133)
Supplement: Supplemental Figure 1: [file tom-00133-16-s001.pdf]

Supplementary Figure 1:

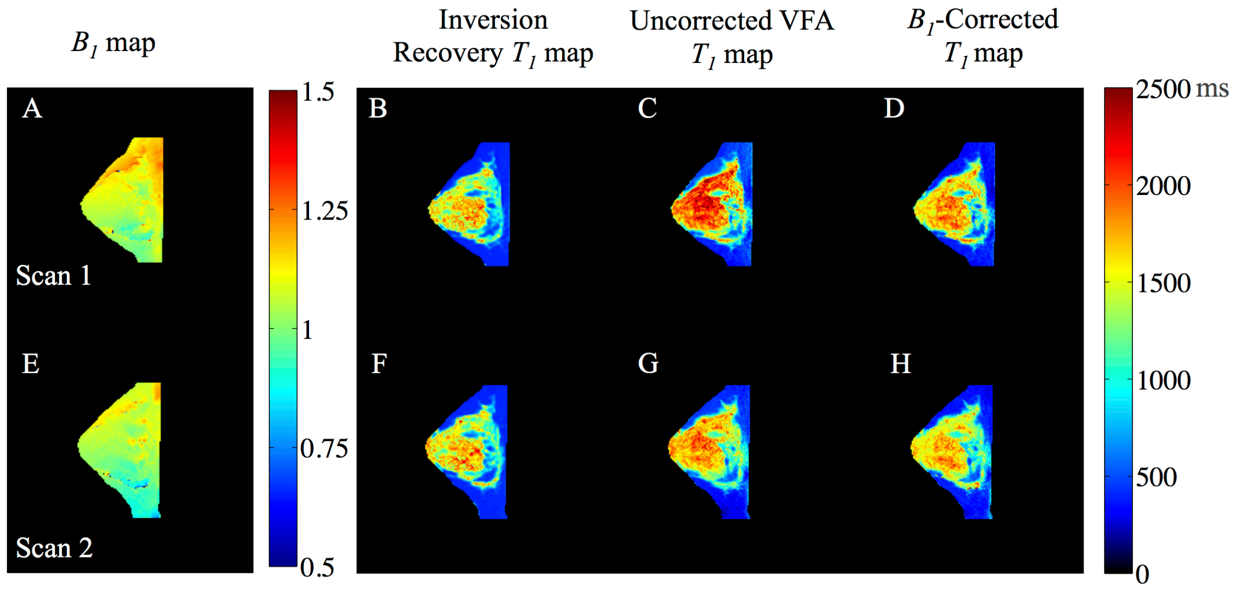

Supplemental Figure 1. A representative test-retest set of B_1 and T_1 parametric maps displayed for the right breast of a healthy volunteer. Bloch-Siegert B_1 maps (A and E) correspond to the correction between the actual and the nominal flip angles. Similar to Figure 2, note the spatial variation of the correction factors in the B_1 maps and the difference in B_1 maps between repeated scans. Thus, again providing evidence that a B_1 map should be incorporated into routine breast imaging. T_1 parametric maps include: IR maps (B and F), uncorrected VFA maps (C and G), and B_1-corrected VFA maps (D and H). Similarly to the right breast (Figure 2), the observed spatial variations in T_1 of the FGT are minimized after B_1 correction so that the T_1 map more closely matches the IR T_1 map. Furthermore, the B_1-corrected T_1 maps are visually more similar between repeated measurements compared to the uncorrected data.
