# Supplementary material for: Bloch–Siegert B1-Mapping Improves Accuracy and Precision of Longitudinal Relaxation Measurements in the Breast at 3 T
Source: Tomography. 2016 Dec;2(4):250–9. doi: 10.18383/j.tom.2016.00133 (PMC5201175; doi:10.18383/j.tom.2016.00133)
Supplement: Supplemental Figure 2: [file tom-00133-16-s002.pdf]

Supplementary Figure 2:

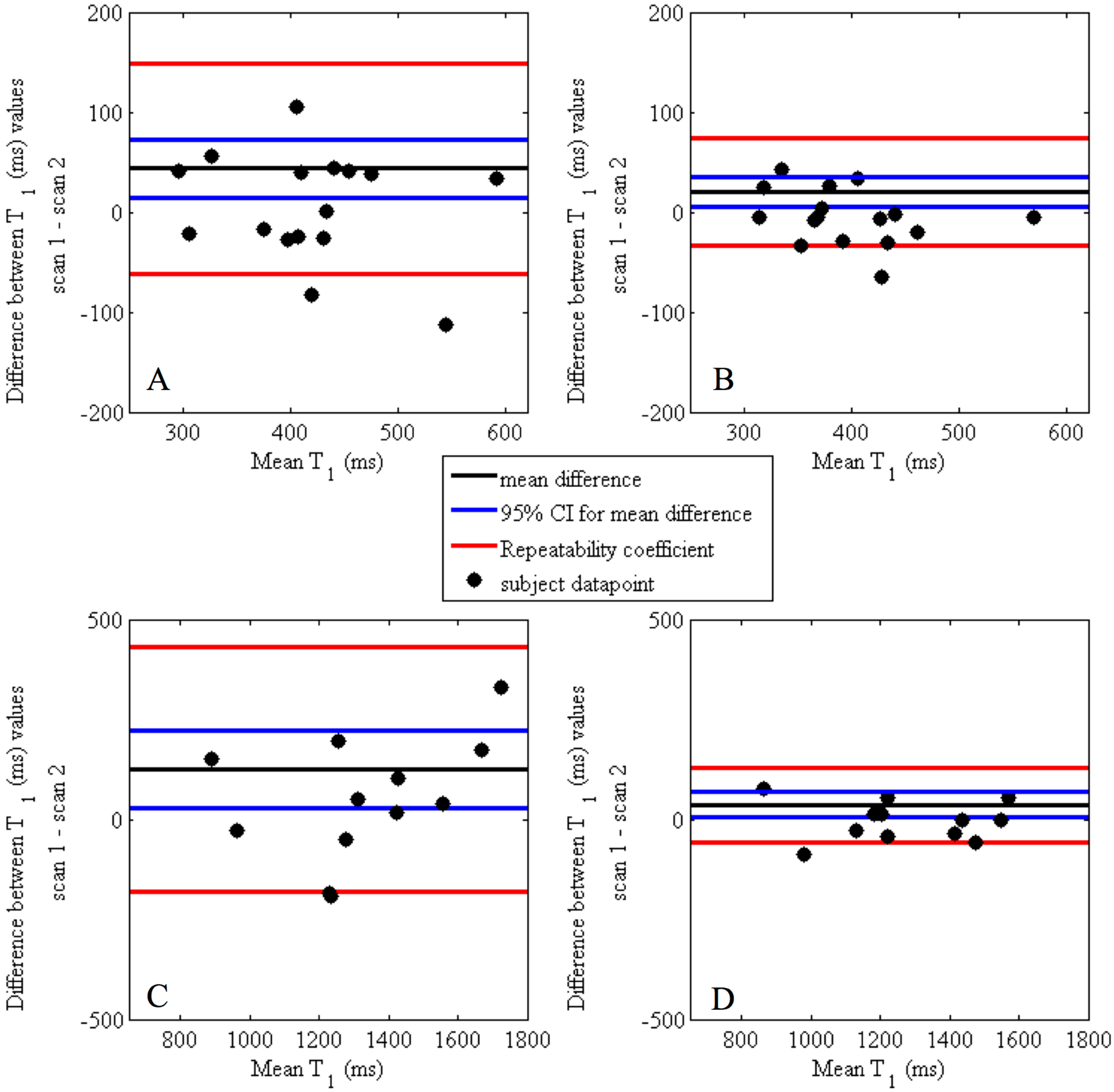

Supplemental Figure 2. Bland-Altman plots for the left breast displaying the difference in T_1 between repeated measurements plotted against mean T_1 for A) AT before B_1 correction, B) AT after B_1 correction, C) FGT before B_1 correction, and D) FGT after B_1 correction. The mean difference (black line) is shown with 95% confidence intervals of the mean difference (blue lines), which defines a measure of the spontaneous variability that is expected in a cohort of subjects. Repeatability is also shown (red lines), which quantifies the maximum difference expected to be observed between two repeat measurements in an individual. Similarly to Figure

4, the width of both the 95% CIs of the mean difference and repeatability coefficient decrease after B_1 correction thus suggesting lower variability.
