# Supplementary material for: Bloch–Siegert B1-Mapping Improves Accuracy and Precision of Longitudinal Relaxation Measurements in the Breast at 3 T
Source: Tomography. 2016 Dec;2(4):250–9. doi: 10.18383/j.tom.2016.00133 (PMC5201175; doi:10.18383/j.tom.2016.00133)
Supplement: Supplemental Figure 3: [file tom-00133-16-s003.pdf]

Supplementary Figure 3:

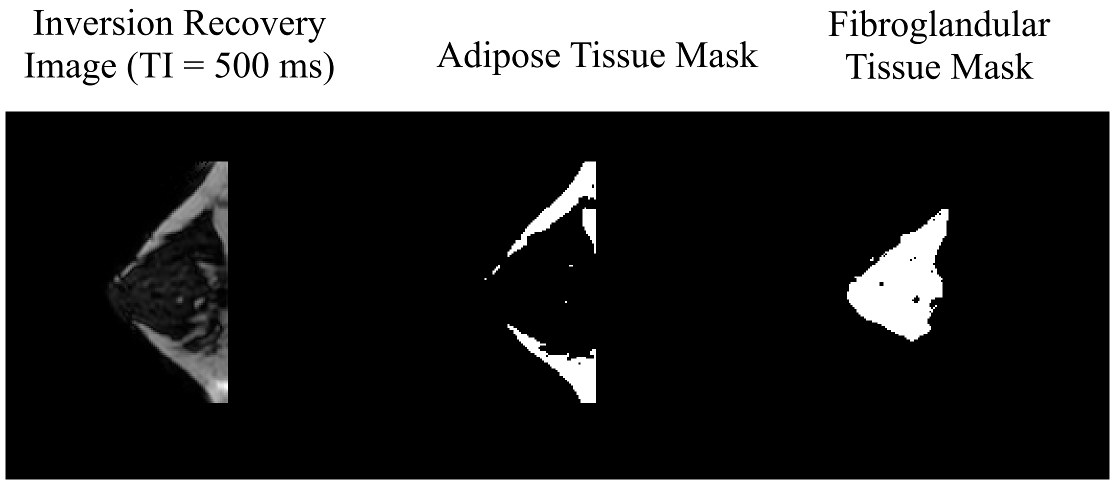

Supplemental Figure 3. A representative example of the segmentation masks used for the analysis; these presented here correspond to parametric maps generated from scan 1 of Figure 2. The inversion recovery image at TI of 500 ms was used to manually segment the AT as the signal intensity from the FGT was close to zero. The FGT mask was subsequently generated as the complement of the AT mask after manually segmenting the skin and chest wall from the FOV.
