# Supplementary material for: Bloch–Siegert B1-Mapping Improves Accuracy and Precision of Longitudinal Relaxation Measurements in the Breast at 3 T
Source: Tomography. 2016 Dec;2(4):250–9. doi: 10.18383/j.tom.2016.00133 (PMC5201175; doi:10.18383/j.tom.2016.00133)
Supplement: Supplemental Table 1: [file tom-00133-16-s004.pdf]

Supplementary Table 1:

**Supplemental Table 1.** Average T_1 values from the right breast of each healthy volunteer												
	**Scan 1: mean T_1 in adipose tissue**			**Scan 1: mean T_1 in fibroglandular tissue**			**Scan 2: mean T_1 in adipose tissue**			**Scan 2: mean T_1 in fibroglandular tissue**		
**Subject #**	**IR**	**VFA w/o B_1**	**VFA w/B_1**	**IR**	**VFA w/o B_1**	**VFA w/B_1**	**IR**	**VFA w/o B_1**	**VFA w/B_1**	**IR**	**VFA w/o B_1**	**VFA w/B_1**
TR_01	494	405	453	1308	1055	1355	461	528	462	1300	1348	1453
TR_02	400	364	383	1221	1185	1281	416	384	389	1228	1193	1250
TR_03	413	255	381	1413	1230	1531	422	303	384	1442	1463	1473
TR_04	417	424	416	1187	1467	1360	423	412	428	1167	1338	1262
TR_05	408	419	400	1261	1589	1320	410	385	407	1198	1384	1388
TR_06	404	435	420	1047	1300	1174	402	432	388	1088	1296	1194
TR_07	401	372	385	N/A	N/A	N/A	397	433	420	N/A	N/A	N/A
TR_08	479	573	525	792	873	851	475	592	500	775	876	799
TR_09	397	337	347	N/A	N/A	N/A	400	441	413	N/A	N/A	N/A
TR_10	437	492	495	1191	1532	1214	441	475	459	1263	1387	1262
TR_11	390	367	374	900	1196	1175	396	396	384	942	1158	1155
TR_12	388	547	397	N/A	N/A	N/A	391	455	380	N/A	N/A	N/A
TR_13	413	444	421	1450	1734	1533	415	434	406	1428	1701	1489
TR_14	425	521	486	1180	1477	1222	428	486	471	1203	1404	1223
TR_15	410	451	450	970	1150	1058	413	452	441	946	1256	1112
TR_16	420	337	345	N/A	N/A	N/A	422	361	351	N/A	N/A	N/A

IR inversion recovery, *VFA* variable flip angle
