# Supplementary material for: Bloch–Siegert B1-Mapping Improves Accuracy and Precision of Longitudinal Relaxation Measurements in the Breast at 3 T
Source: Tomography. 2016 Dec;2(4):250–9. doi: 10.18383/j.tom.2016.00133 (PMC5201175; doi:10.18383/j.tom.2016.00133)
Supplement: Supplemental Table 2: [file tom-00133-16-s005.pdf]

Supplementary Table 2:

Supplemental Table 2. Average T_1 values from the right breast of each healthy volunteer

	Scan 1: mean T_1 in adipose tissue			Scan 1: mean T_1 in fibroglandular tissue			Scan 2: mean T_1 in adipose tissue			Scan 2: mean T_1 in fibroglandular tissue		
**Subject #**	**IR**	**VFA w/o B_1**	**VFA w/B_1**	**IR**	**VFA w/o B_1**	**VFA w/B_1**	**IR**	**VFA w/o B_1**	**VFA w/B_1**	**IR**	**VFA w/o B_1**	**VFA w/B_1**
TR_01	437	495	439	1439	1479	1400	457	456	441	1387	1374	1432
TR_02	395	378	362	1088	1139	1116	395	461	370	1126	1323	1143
TR_03	403	475	378	1449	1759	1549	409	433	407	1462	1582	1550
TR_04	417	488	396	1133	1430	1212	411	600	461	1292	1412	1197
TR_05	410	459	423	1281	1890	1597	415	353	389	1377	1557	1541
TR_06	406	430	393	1050	949	936	404	390	366	1075	977	1022
TR_07	404	317	332	N/A	N/A	N/A	401	275	306	N/A	N/A	N/A
TR_08	487	609	568	803	967	905	479	575	572	810	814	825
TR_09	397	355	357	N/A	N/A	N/A	391	298	313	N/A	N/A	N/A
TR_10	442	418	418	1280	1575	1436	434	443	448	1307	1535	1437
TR_11	394	384	366	1027	1253	1190	399	411	371	1031	1300	1175
TR_12	394	395	337	N/A	N/A	N/A	401	419	370	N/A	N/A	N/A
TR_13	429	368	424	1277	1139	1444	436	384	430	1408	1330	1502
TR_14	433	463	452	1102	1353	1201	452	418	472	1141	1156	1241
TR_15	414	434	375	1103	1338	1249	418	433	370	1160	1287	1191
TR_16	419	296	313	N/A	N/A	N/A	426	317	317	N/A	N/A	N/A

IR inversion recovery, *VFA* variable flip angle
