# Supplementary material for: Bloch–Siegert B1-Mapping Improves Accuracy and Precision of Longitudinal Relaxation Measurements in the Breast at 3 T
Source: Tomography. 2016 Dec;2(4):250–9. doi: 10.18383/j.tom.2016.00133 (PMC5201175; doi:10.18383/j.tom.2016.00133)
Supplement: Supplemental Table 3: [file tom-00133-16-s006.pdf]

Supplementary Table 3:

Supplemental Table 3. Reproducibility results for the left breast

	**Mean**	**Mean difference**	**95% CI for mean difference**	**wSD**	**Repeatability**	**CV (mean ± SD)**
Adipose Tissue						
VFA	420	45	±29 (6.9%)	38	105	7.6% ± 5.0%
VFA w/B_1	398	21	±15 (3.8%)	19	54	3.9% ± 3.3%
Fibroglandular tissue						
VFA	1330	127	±98 (7.4%)	111	306	5.5% ± 4.7%
VFA w/B_1	1270	39	±32 (2.5%)	34	94	2.2% ± 1.9%

CI confidence interval, *wSD* within-subject standard deviation, *CV* coefficient of variation
